# Perturbation of mammary epithelial cell apicobasal polarity by RHBDF1-facilitated nuclear translocation of PKCζ

**DOI:** 10.1186/s40659-024-00566-2

**Published:** 2024-11-24

**Authors:** Huan-Yu Zhao, Yi-Pan Zhu, Ying Wen, Jing Sun, Xin-Yu Ding, Xin-Yu Cao, Kai-Liang Wu, Li Fu, Lu-Yuan Li

**Affiliations:** 1 State Key Laboratory of Medicinal Chemical Biology and College of Pharmacy, The Haihe Laboratory of Cell Ecosystem, Tianjin Key Laboratory of Molecular Drug Research, Nankai University, Tianjin, 300350 China; 2 Department of Pharmacology, Second Military Medical University/Naval Medical University, Shanghai, 200433 China; 3 Department of Breast Cancer Pathology and Research Laboratory, Cancer Institute and Hospital, Tianjin Medical University, Tianjin Medical university, Tianjin, 300060 China

**Keywords:** RHBDF1, PKCζ, Apicobasal polarity, Tight junction, Adherens junction, Cell invasion

## Abstract

**Background:**

The establishment of apicobasal polarity in epithelial cells is of critical importance in morphogenesis of mammary gland and other secretive gland tissues. The demise of the polarity is a critical step in early stages of tumorigenesis such as in breast ductal carcinoma in situ. The underlying molecular mechanism thus warrants in-depth investigations.

**Results:**

Protein kinase C isoform ζ (PKCζ), which is highly expressed in breast cancer cells, accumulates in the nuclei of human mammary epithelial cells overexpressing human rhomboid family-1 (RHBDF1), an endoplasmic reticulum membrane protein. Nuclear translocation of PKCζ results in the failure of the formation of the cytosolic apicobasal polarity complex Par, of which PKCζ is an essential component. Additionally, enhanced nuclear translocation of PKCζ is accompanied by an inhibition of the expression of cell tight junction and adherens junction proteins and an increase of cell mobility. Mechanistically, RHBDF1 is able to interact with importin β1 and PKCζ and promote PKCζ phosphorylation. Consistently, treatment of RHBDF1-overexpressing cells with an inhibitor of PKCζ phosphorylation leads to restoration of apicobasal polarity and cell-cell junctions, as well as suppressed cell mobility.

**Conclusions:**

RHBDF1-facilitated nuclear translocation of PKCζ is critically responsible for the dismantlement of epithelial cell apicobasal polarity, and thus may serve as a target in the development of therapeutic approaches against early stages of breast cancer.

**Supplementary Information:**

The online version contains supplementary material available at 10.1186/s40659-024-00566-2.

## Introduction

The human rhomboid family-1 protein (RHBDF1, also known as iRhom1) is a multiple-transmembrane protein mainly found in the endoplasmic reticulum and Golgi apparatus [1, 2]. RHBDF1 gene expression is present marginally in normal tissues, but is highly prominent in clinical specimens of breast cancer [3, 4]. Silencing the RHBDF1 gene in experimental tumor models leads to apoptosis and autophagy in breast cancer and neck squamous cell cancer cells and inhibition of tumor growth [4]. The function of the RHBDF1 protein appears to be multifaceted, including facilitation of TGFα secretion and thus mediating G-protein coupled receptor (GPCR) ligand-activated transactivation of epidermal growth factor receptor (EGFR) [5, 6], protection of oxygen-independent degradation of hypoxia-inducible factor-1α (HIF-1α) [3], and promotion of endothelial-mesenchymal transition by activating the JNK/AP-1 signaling pathway, giving rise to destabilization of blood vessels, intensified hypoxic and inflammatory conditions, and augmented fibrotic stroma formation in breast cancer [7]. The various and important functions of RHBDF1 make this protein an attractive subject for cancer research.

We have shown that RHBDF1 takes part in the disruption of the establishment of apicobasal polarity in mammary gland epithelial cells [8]. The establishment of apicobasal polarity in epithelial cells is of critical importance in morphogenesis of mammary gland and other secretive gland tissues [9, 10]. Loss of apicobasal polarity and epithelial barrier such as the tight junction (TJ) and adherens junction (AJ) are characteristic of epithelial cell carcinogenesis [11, 12]. It has been shown that polar complexes Par, Crumbs and Scrib play a decisive role in the establishment and maintenance of epithelial apicobasal polarity and gland morphogenesis [13, 14]. The Par complex has a component known as atypical protein kinase C (aPKC, PKCζ/ι), which has the ability to catalyze the phosphorylation of members of the other two complexes such as Lgl and Crb3 [14–17]. The molecular mechanism underlying the disruption of apicobasal polarity and the impairment of epithelial barrier by the action of RHBDF1, therefore, warrants in-depth investigations.

In the study we show that RHBDF1 is involved in the translocation of PKCζ from the cytosol to the nucleus. Diminished PKCζ protein levels in the cytosol result in a disruption of epithelial apicobasal polarity and TJ/AJ barrier. These findings provide insights into the molecular mechanisms underlying the failure of the maintenance of epithelial gland morphology, which often takes place in early stages of breast carcinogenesis.

## Results

### RHBDF1-overexpression in mammary epithelial cells leads to elevated-invasion and downregulated TJ/AJ markers

**Fig. 1:**
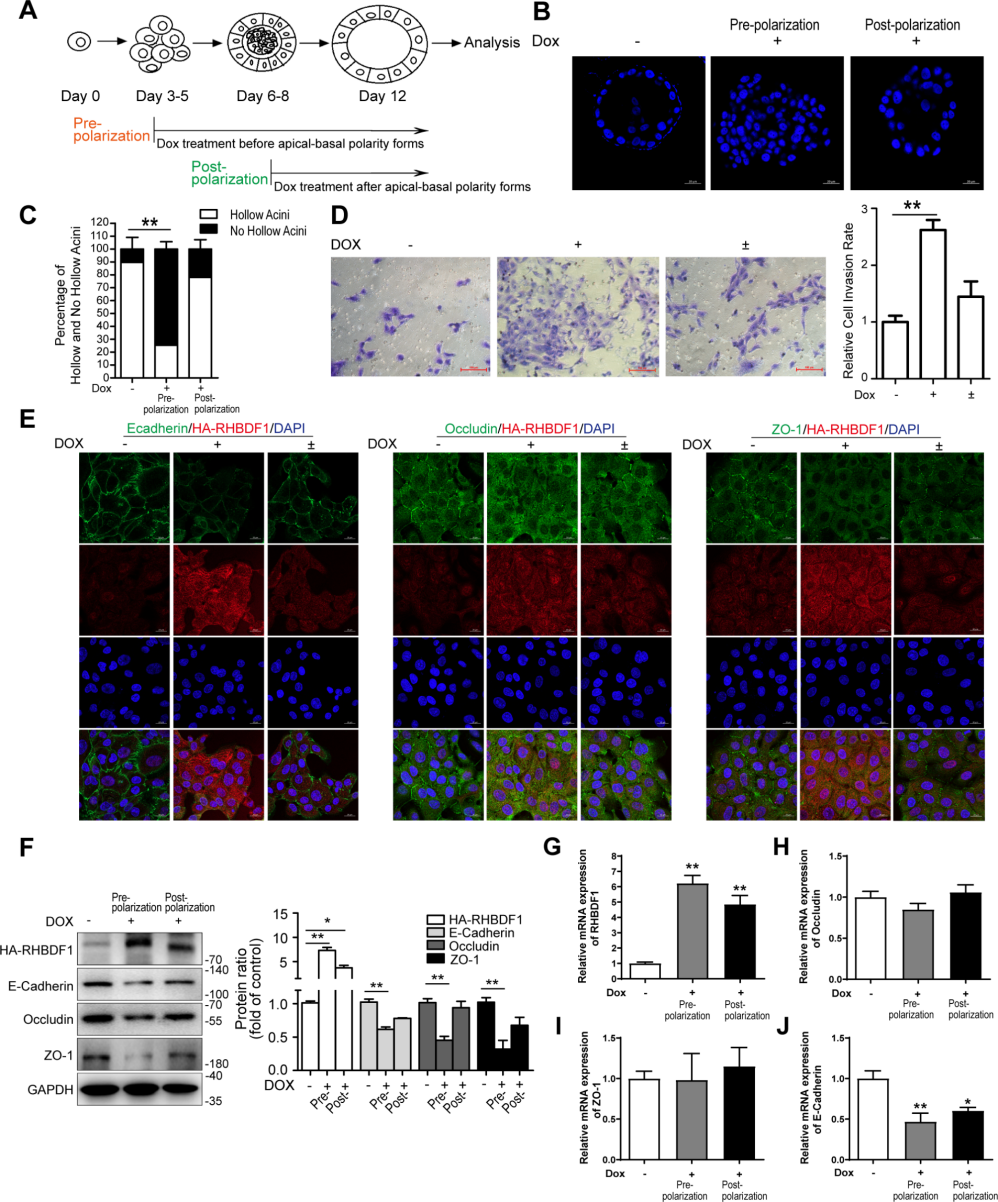
RHBDF1-overexpression in mammary epithelial cell leads to elevated-invasion rate and downregulated TJ/AJ markers. (**A**) Experimental scheme for the induction of RHBDF1 by Dox in acini before and after polarization. (**B**) The confocal images of DAPI-stained equatorial cross sections of TetON-RH acini treated with Dox before and after polarization (Scale bar, 20 μm). (**C**) Quantification of the percentage of the hollowed/no hollowed acini in TetON-RH acini (*n* = 100). (**D**) Typical images of invasion of Dox (2 µg/mL) treated TetON-RH cells for 96 h, or then removed Dox for another 96 h; scale bar, 100 μm; (-) was solvent control for 96 h (Dox-off); (+) was Dox treated for 96 h (Dox-on); (±) was treated by Dox for 96 h, then removed and cultured for another 96 h (Dox-on/off); bar graphs show the ratio of invasion cells (*n* = 3). (**E**) Typical images of E-cadherin, Occludin or ZO-1 with RHBDF1 immunofluorescence-stained TetON-RH cells; scale bar 20 μm (*n* = 3). (**F**) The protein expression of HA-RHBDF1, E-cadherin, Occludin, ZO-1 in Dox stimulated TetON-RH in 3D acini cultures (*n* = 3). (**G**-**J**) The mRNA expression of RHBDF1 (**G**), Occludin (**H**), ZO-1 (**I**) and E-Cadherin (**J**) in Dox stimulated TetON-RH in 3D acini cultures (*n* = 3). These experiments were repeated two times under identical conditions. Data are mean ± SD, ^**^P<0.01, ^*^P<0.05, data in (**C**) is analyzed by two-way ANOVA; others are analyzed by one-way ANOVA

We artificially overexpressed RHBDF1 in epithelial cells of non-tumorous human MCF10A cell-line under tetracycline (Dox) regulation (TetON-RH). The cells were cultured on a thick layer of extracellular membrane proteins (Matrigel) in the absence or presence of Dox. The transfected RHBDF1 gene was silent in the absence of Dox, and the cells were able to form acini, each with a lumen, indicating that the cells were able to establish apical-basal polarity (Fig. 1A-C) under the experimental conditions. In the presence of Dox, however, the cells formed solid aggregates. Interestingly, addition of Dox to the culture media post acinus formation did not significantly affect the existing acini (Fig. 1A-C). These data indicate that RHBDF1 overexpression prevent epithelial cell acinus formation in this 3-dimentional (3D) cell culture model.

We then determined the impact of RHBDF1 on cell mobility by measuring the number of cells passing through minipore filters coated with Matrigel. We found that the passing rate of the cells increased by about 2.5 times under RHBDF1 overexpression conditions (Dox-on) compared with Dox-off conditions (Fig. 1D). We thus determined the mechanism of RHBDF1-stimulated epithelial cell mobility by evaluating the expression of E-cadherin, Occludin and ZO-1 that are key proteins of TJ/AJ structures [18–20]. First, we carried out fluorescent immunostaining of the cells to determine the impact of RHBDF1 overexpression on the distribution of the TJ/AJ proteins (Fig. 1E). We found that high levels of RHBDF1 disrupted TJ/AJ formation. We then found that, in the 3D acinus formation model under Dox-on conditions, as RHBDF1 protein levels in the cells increased by about 9 times in 96 h, the protein levels of E-cadherin, Occludin and ZO-1 declined by about 40%, 50% and 70%, respectively, in comparison with that under Dox-off conditions (Fig. 1F). When Dox was withdrawn and the cell cultures maintained for another 96 h (Dox±), RHBDF1 protein levels decreased substantially, while that of E-cadherin, Occludin and ZO-1 protein levels were restored to levels similar to those under Dox-off conditions (Fig. 1F). At the same time, we found by qPCR analyses that mRNA levels of Occludin and ZO-1 were unaffected by RHBDF1 overexpression (Fig. 1G-I) whereas E-cadherin mRNA level decreased by about 50% when RHBDF1 was overexpressed (Fig. 1J). These findings indicate that RHBDF1-promoted cell mobility is associated with a simultaneous decrease of the TJ/AJ proteins.

### RHBDF1 facilitates PKCζ activation and accumulation in cell nucleus

**Fig. 2:**
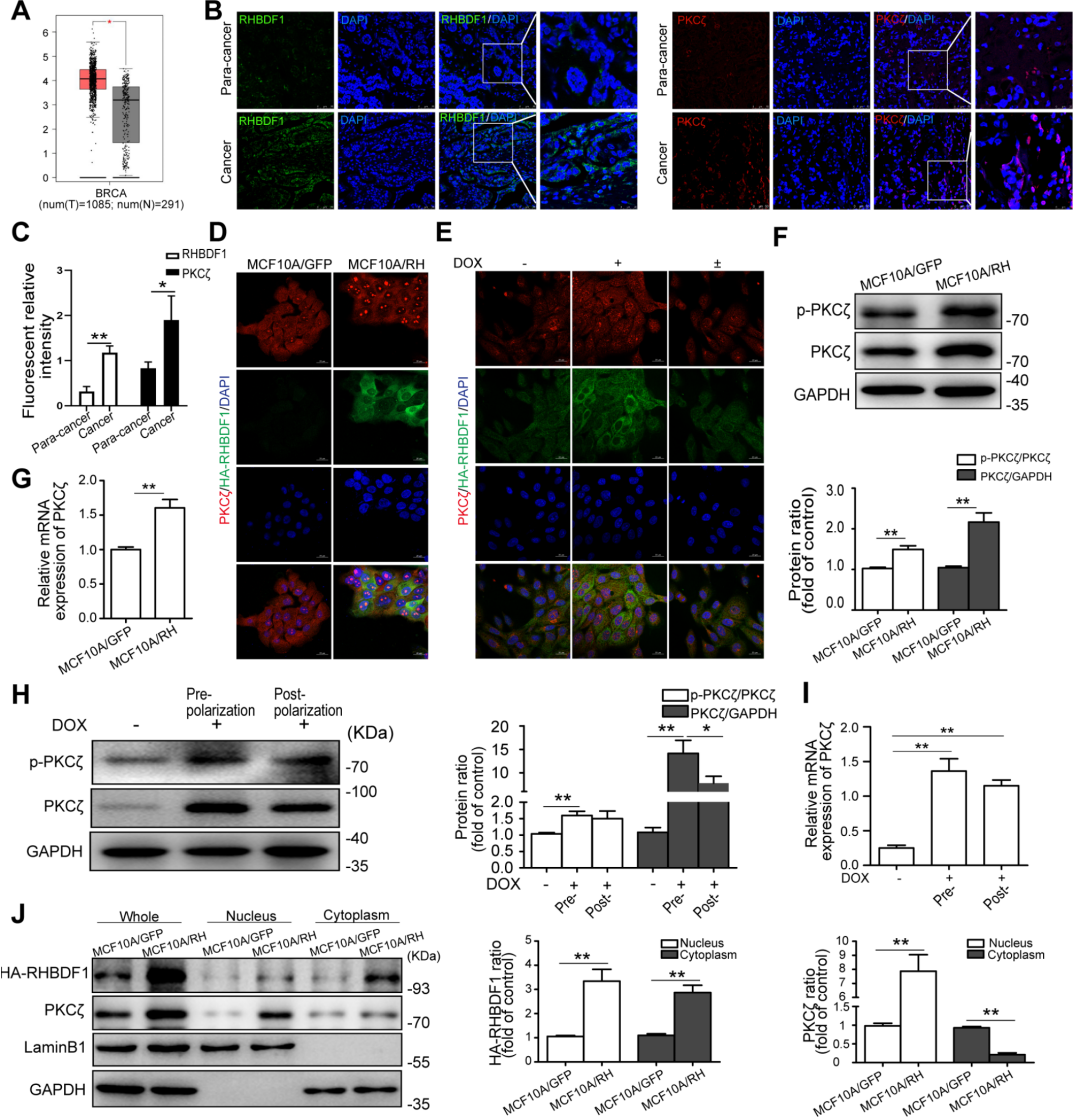
RHBDF1 facilitates PKCζ activation and accumulation in cell nucleus. (**A**) Boxplot of the expression of PKCζ in breast cancer from TCGA database. N: Normal tissue; T: Tumor tissue. (**B**) Typical images of RHBDF1 (Left) and PKCζ (Right) immunofluorescence-staining of human breast cancer and para-cancerous tissues; scale bar 50 μm (number of patients *n* = 6). (**C**) The statistical analysis of fluorescence density of RHBDF1 and PKCζ in (B). (**D**, **E**) Typical images of PKCζ with RHBDF1 immunofluorescence-stained (**D**) MCF10A/GFP and MCF10A/RH, and (**E**) Dox-stimulated TetON-RH cells; scale bar 20 μm (*n* = 3). (**F**-**I**) The (**F**) protein and (**G**) mRNA levels of p-PKCζ and PKCζ in (**F**, **G**) MCF10A/GFP and MCF10A/RH, and (**H**, **I**) Dox-treated TetON-RH in 3D acini cultures (*n* = 3). (**J**) The levels of HA-RHBDF1 and PKCζ in nucleus and cytoplasm of MCF10A/GFP and MCF10A/RH cells (*n* = 3). These experiments were repeated two times under identical conditions. Data are mean ± SD, ^**^P<0.01, ^*^P<0.05, data in (**G**) and (**I**) are analyzed by one-way ANOVA, others are analyzed by Student-t test

Since protein kinase C-ζ (PKCζ) is a critical component of the apicobasal polarity complex of mammary epithelial cells [21, 22], and is highly expressed in breast cancer cells in the TCGA database (*P* < 0.01) (Fig. 2A), we investigated RHBDF1 and PKCζ protein levels in human breast cancer tissues and cancer-adjacent normal tissues (para-cancerous tissues). Fluorescent immunostaining showed that both PKCζ and RHBDF1 protein levels were higher in cancer tissues (Fig. 2B, C). Interestingly, there was a considerable amount of the PKCζ protein in the nuclei in the cancer tissues compared to that in para-cancerous tissues (Fig. 2B). Additionally, a significant amount of the PKCζ protein accumulated in the nuclei of RHBDF1-overexprssing MCF10A cells (MCF10A/RH) (Fig. 2D) as well as in TetON-controlled RHBDF1 overexpression cells (Fig. 2E). We further found that MCF10A/RH cells in the 3D acinus formation model showed an about 1.5 times increase of p-PKCζ and an about 2 times increase of total PKCζ protein (Fig. 2F), and the PKCζ mRNA levels increased about 1.5 times (Fig. 2G), compared to that in MCF10A/GFP control cells. Additionally, in the 3D model of TetON-controlled RHBDF1 overexpression cells, p-PKCζ protein levels increased by about 1.5 times and the total PKCζ protein levels increased by about 13 times (Fig. 2H). The PKCζ mRNA levels also increased by about 5.2 times and 4.8 times, respectively, at the pre-polarization stage and the post-polarization stage (Fig. 2I), in comparison with the results under TetOff conditions. Moreover, we determined the distribution of the PKCζ protein in the cells isolated from the 3D cultures, and found that it was present predominantly in the nucleus fragment of the MCF10A/RH cells (Fig. 2J). These findings suggest that RHBDF1 be able to facilitate the translocation of PKCζ from the cytoplasm to the nucleus of the epithelial cells in the 3D model.

### Blocking PKCζ phosphorylation leads to suppression of RHBDF1-facilitated disruption of cell polarity and TJ/AJ functions

**Fig. 3:**
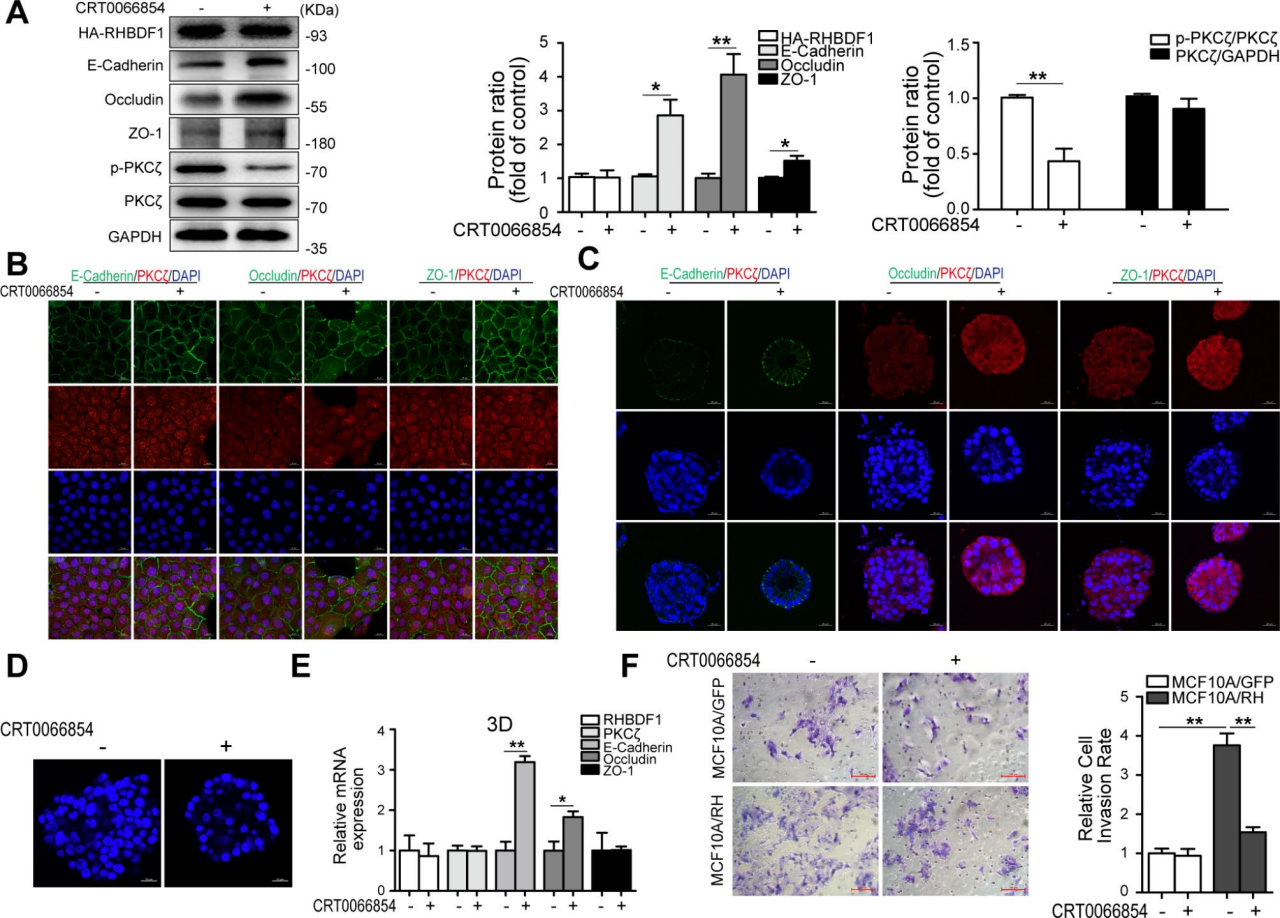
Blocking PKCζ phosphorylation leads to suppression of RHBDF1-facilitated disruption of cell polarity and TJ/AJ functions. (**A**) The expression of related proteins in MCF10A/RH acini with CRT0066854 (4 µM) stimulation on the day 4 and cultured for another 6 days in 3D cultures. (**B**, **C**) Typical images of E-cadherin, Occludin or ZO-1 with PKCζ immunofluorescence-stained MCF10A/RH with CRT0066854 treatment; scale bar 20 μm (*n* = 3). (**D**) The confocal images of DAPI-stained equatorial cross sections of MCF-10 A/RH acini with CRT0066854; scale bar 20 μm (*n* = 3). (**E**) The mRNA expression levels of related genes in CRT0066854 treated MCF10A/RH for 3D cultures. (**F**) Typical images of invasion for CRT0066854 stimulated MCF10A/GFP and MCF10A/RH cells; scale bar 100 μm; bar graphs show the ratio of invasion cells (*n* = 3). These experiments were repeated two times under identical conditions. Data are mean ± SD, ^**^P<0.01, ^*^P<0.05, data are analyzed by Student-t test

We used a relatively specific inhibitor of PKCζ, CRT0066854 [23], to determine the effect on RHBDF1-facilitated TJ/AJ destruction. We found that the inhibitor was able to inhibit PKCζ activation (p-PKCζ) without affecting PKCζ or RHBDF1 expression in MCF10A/RH cells (Fig. 3A). Treatment of MCF10A/RH cells with CRT0066854 resulted in increases in the protein levels of E-cadherin, Occludin, and ZO-1 by about 3, 4 and 1.5 times, respectively, compared to that in untreated controls (Fig. 3A). PKCζ phosphorylation (p-PKCζ) was also inhibited by similar treatment of the 3D cultures (Fig. 3A). Consistently, fluorescent immunostaining of MCF10A/RH cell cultures for E-cadherin, Occludin, and ZO-1 revealed that CRT0066854 treatment led to increased levels of these TJ/AJ proteins at the cell junctions (Fig. 3B, C). Moreover, CRT0066854 treatment promoted acinus formation (Fig. 3D). In 3D cultures of MCF10A/RH cells, CRT0066854 treatment led to enhanced expression of both E-cadherin and Occludin, but not ZO-1 (Fig. 3E), despite of diminished RHBDF1 levels. Furthermore, we found that CRT0066854 treatment inhibited the rate of MCF10A/RH cell invasion by about 60%, while it had no impact on the invasion rate of the control MCF10A/GFP cells (Fig. 3F). These data demonstrate that inhibition of PKCζ results in a block of RHBDF1-facilitated PKCζ phosphorylation and the ability of RHBDF1 to disrupt epithelial cell polarity and TJ/AJ functions.

### RHBDF1-importin β1 interaction is needed for PKCζ nuclear translocation

**Fig. 4:**
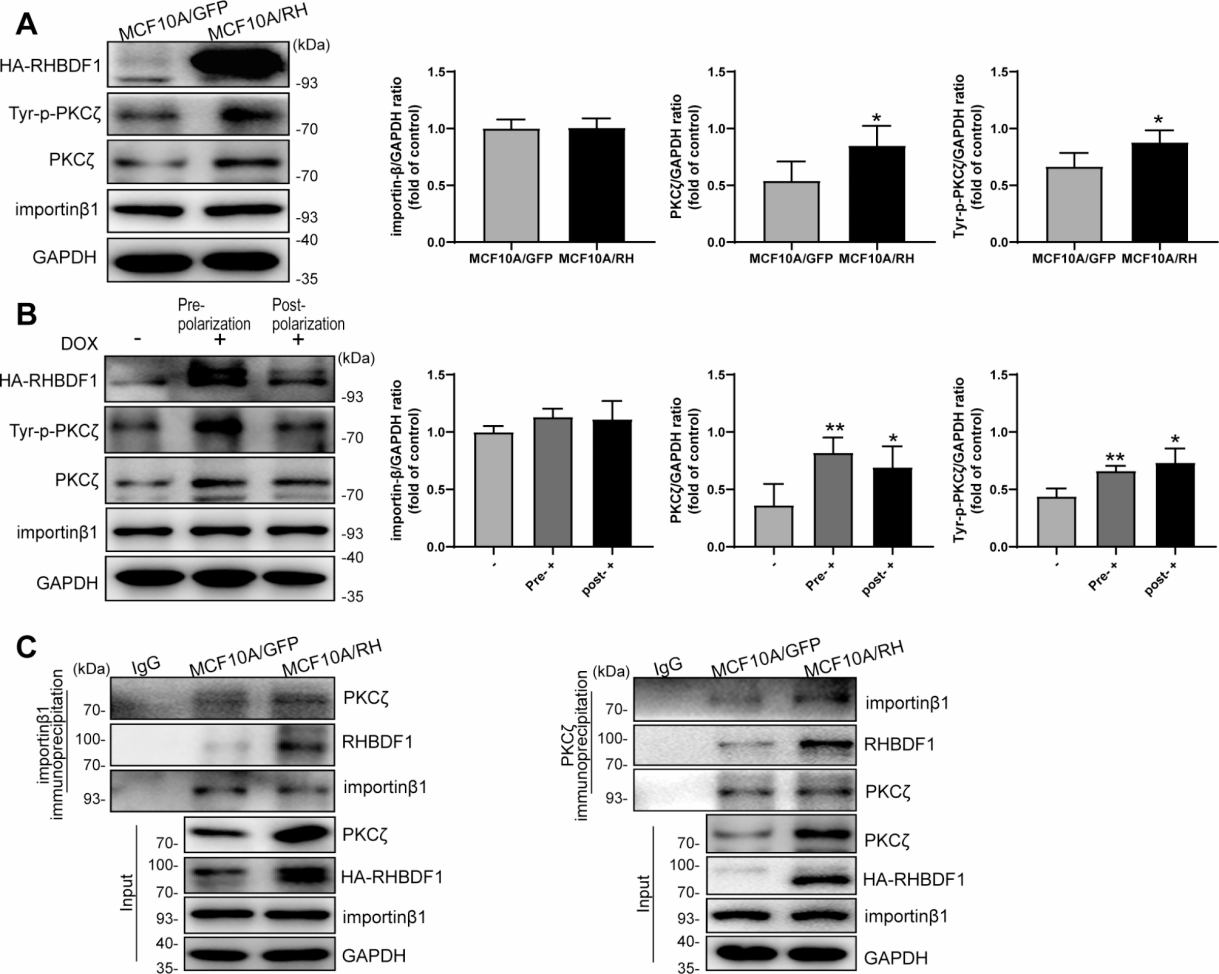
RHBDF1-importin β1 interaction is needed for PKCζ nuclear translocation. (**A**, **B**) The Tyrosine phosphorylation of PKCζ (Tyr-p-PKCζ) and importin β1 protein expression in (A) MCF10A/GFP, MCF10A/RH and (**B**) Dox-treated TetON-RH cells/acini (*n* = 3). (**C**) Co-immunoprecipitation (Co-IP) analysis of the interaction between RHBDF1, importin β1 and PKCζ in MCF10A/GFP and MCF10A/RH under 3D cultures (*n* = 3). These experiments were repeated two times under identical conditions. Data are mean ± SD, data are analyzed by Student-t test

It has been reported previously that the nuclear transporter receptor importin β1 is pivotal in nuclear translocation of aPKC, and that the action depends on aPKC tyrosine phosphorylation [24]. We investigated whether a similar mechanism was applicable in RHBDF1-promoted PKCζ translocation into the nucleus. We found that, although importin β1 protein levels were basically unaffected by RHBDF1 overexpression in MCF10A/RH cells in the acinus formation model by Western blotting analyses (Fig. 4A), the extent of PKCζ tyrosine phosphorylation in these cells were significantly higher than that in the MCF10A/GFP controls (Fig. 4A). We further found that both PKCζ and Tyr-p-PKCζ protein levels became markedly highly elevated once RHBDF1 overexpression was turned on by Dox treatment of the TetON-RH cells either before or after acinus formation in the 3D cultures (Fig. 4B). We then carried out co-immunoprecipitation (co-IP) experiments and found that importin β1 was able to co-IP simultaneously with both PKCζ and RHBDF1. Likewise, PKCζ was able to co-IP simultaneously with both importin β1 and RHBDF1 under these experimental conditions (Fig. 4C). These findings are consistent with the view that a RHBDF1-importin β1-PKCζ protein complex is involved in RHBDF1-facilitated nuclear translocation of PKCζ.

## Discussion

Three-dimensional cellular models are increasingly becoming a key tool for studying cancer cell behaviors because of the ability of these models to simulate a set of circumstances in vivo under given experimental conditions [25, 26]. In this study, we used a 3D cell culture model of human mammary epithelial cells from non-tumorous cell line MCF10A. We show in this study that RHBDF1-promoted, importin β1-assisted, transportation of PKCζ from the cytosol to the nucleus is likely attributable to the disruption in the formation of the apicobasal polarity complex, of which PKCζ is a key component [21, 27]. Additionally, the collapse of cell polarity is accompanied by downregulated cell-cell TJ/AJ formation which, in turn, results in augmented cell mobility. Mechanistically, RHBDF1 not only promotes tyrosine-phosphorylation of PKCζ, but also interacts with Tyr-p-PKCζ and importin β1 to facilitate the translocation of PKCζ into the nucleus. The findings support the view that RHBDF1 overexpression, as it is often observed in clinic breast cancer tissues, may facilitate the loss of apicobasal polarity in mammary gland epithelial cells during early stages of breast tumorigenesis.

**Fig. 5:**
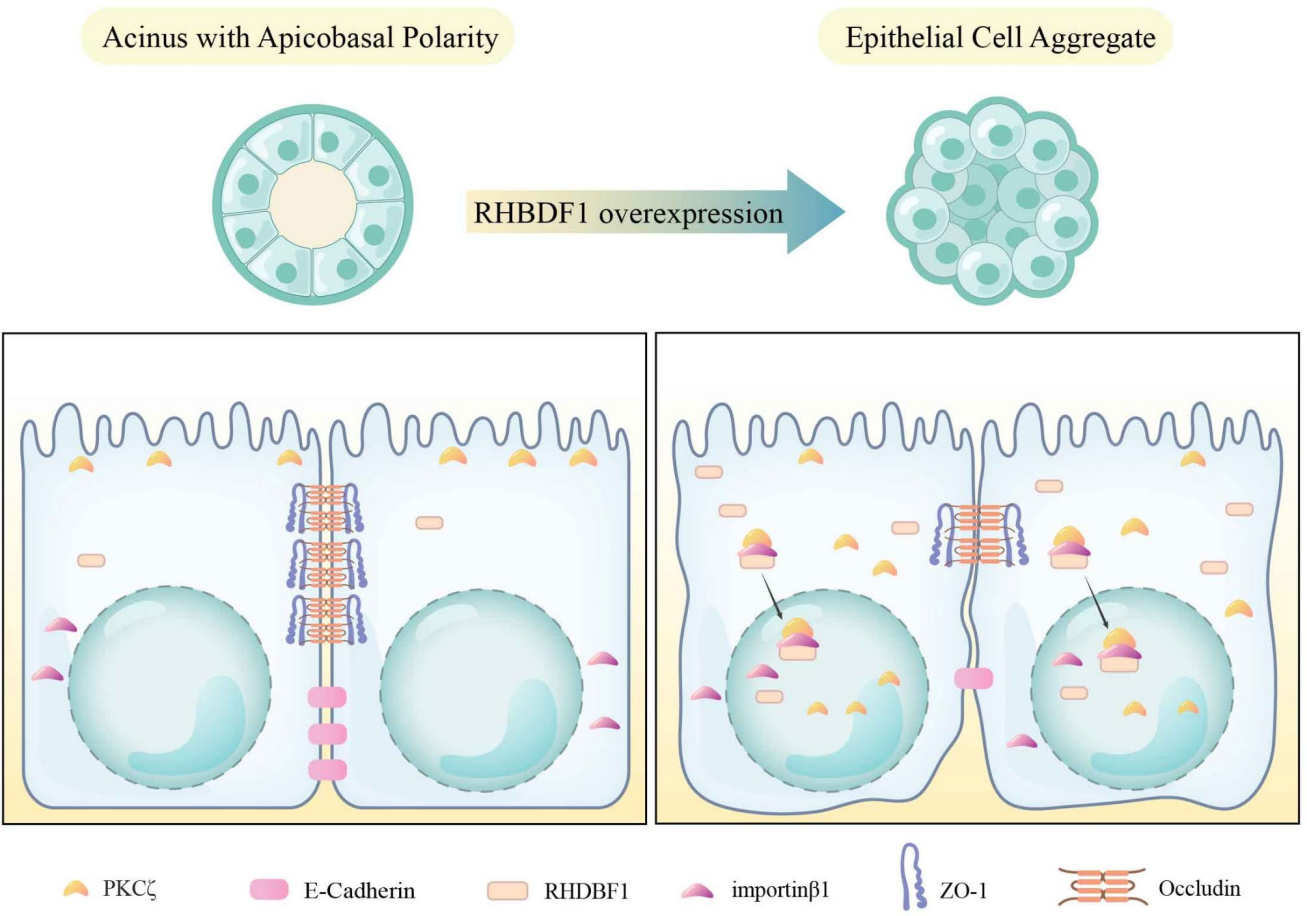
Schematic representation of RHBDF1-promoted destruction of mammary epithelial cell apicobasal polarity and cell-cell connections

Based on our findings from these cell culture models, we propose that the molecular mechanism underlying RHBDF1-driven demise of mammary epithelial apicobasal polarity consists of two parts (Fig. 5). The first part is RHBDF1-stimulated activation of PKCζ and importin β1-assisted translocation of activated PKCζ. The second part is the downregulation of TJ/AJ proteins, namely Occludin, ZO-1 and E-cadherin, as a consequence of RHBDF1-facilitated activation of PKCζ. PKCζ is known to be an atypical protein kinase that is part of the PAR complex essential in the maintenance of normal cell polarity. The PAR complex is located on the plasma membrane and regulates apical-basal polarity by stimulating the formation of intercellular connections [28, 29]. The nuclear transport protein importin β1 plays a crucial role in the translocation of cytosolic proteins into the nucleus by recognizing specific nuclear localization sequences, forming nuclear transport complexes, and changing the permeability of the nuclear pore complexes to complete the nuclear translocation of proteins [30–32]. That RHBDF1 forming a protein complex with PKCζ and importin β1 provides a new mechanism supporting material transport between cytosol and nucleus.

Additionally, our data indicate that RHBDF1 overexpression in a 3D model of non-tumorous breast epithelial cell line MCF10A leads to downregulation of Occludin, ZO-1 and E-cadherin, which are essential components of TJ and AJ complexes [33, 34]. Interestingly, ZO-1 is involved in recruiting proteins such as E-cadherin and Occludin to the intercellular junction region [35, 36]. Dynamic changes of TJ/AJ are closely related not only to epithelial polarity, but also to stable intercellular connections for normal material exchanges [10, 37]. Consequently, breakdown of epithelial cell polarity would lead to weakened cell-cell junctions [38, 39], thus allowing disordered cell growth, migration, and invasion. Moreover, our findings are suggestive that nuclear translocation of PKCζ result in certain gene expression regulation, although it remains unclear as per the role of activated PKCζ in the nucleus and the consequent molecular and biological events, which is a point worth of further clarifying.

In summary, our findings indicate that the perturbation of mammary epithelial cell apicobasal polarity is driven at least partly by RHBDF1-facilitated nuclear translocation of PKCζ, and that this process involves the formation of RHBDF1-PKCζ-importin β1 complex. These data suggest that the RHBDF1-PKCζ-importin β1 complex may have a utility to serve as a target for the development of therapeutic approaches aiming at curtailing the disruption of epithelial cell polarity and mammary gland normality in early stage of breast cancer development.

## Materials and methods

### Cell culture and establishment of stable overexpressed RHBDF1 MCF-10 A cell lines

MCF10A cells were purchased from American Type Culture Collection (ATCC, Rockefeller, USA), and cultured in the culture media with 5% CO_2_ at 37 ℃. The culture media formulation is as follows: 500 mL DMEM/F12 (Invitrogen, Carlsbad, USA), containing 5% (v/v) Horse serum (Solarbio, Beijing, China), 20 ng/mL of Recombinant Human EGF (PeproTech, Rocky Hill, USA), 0.5 µg/mL, 100 ng/mL and 10 µg/mL of Hydrocortisone, Cholera toxin and Insulin, respectively (MedChemExpress, New Jersey, USA), and 1% penicillin (50 U/mL)-streptomycin (50 µg/mL) (Invitrogen, Carlsbad, USA).

The lentivirus: pLV[Exp]-EGFP: T2A: Puro-Null, pLV[Exp]-EGFP: T2A: Puro-EF1A > HA/hRHBDF1 and pLV[TetOn]-EGFP: T2A: Puro-TRE > HA/hRHBDF1 used to infect MCF10A cells were purchased from VectorBuilder (Guangzhou, China). To establish stably overexpressing RHBDF1 cells, MCF-10 A cells were plated at 5 × 10^4^ cells per 24-well plate and performed lentivirus infection with MOI 30 and an addition of 5 mg/mL polybrene for 12 h, then in fresh culture medium for 48 h. The stably transduced cells were cultured in the presence of puromycin (0.5 µg/mL) selection for at least 5 days and gained stable overexpressed RHBDF1/HA MCF-10 A (MCF10A/RH) cell lines and its control (MCF10A/GFP), and tetracycline-inducible RHBDF1/tST (TetON-RH/tST) cell lines. Then TetON-RH/tST cells were cultured in the presence of Hygromycin B (20 µg/mL) selection for at least 10 days and gained tetracycline-inducible RHBDF1 (TetON-RH) cell lines. TetON-RH cells were treated with 2 µg/mL Doxycycline (Dox, MedChemExpress) for 96 h to induce overexpression of RHBDF1. The transfection efficiency was monitored by qPCR and Western blot analysis for RHBDF1 expression.

### 3D cell culture

The 3D cell culture model is as previously described [40]. Cells were seeded in Chamber SlidE 8 well Glass/Lab-Tek (Thermo Scientific, Waltham, USA) on Growth Factor reduced Matrigel (BD Biosciences, State of New Jersey, USA) in 2% Matrigel 3D-Assay media with 5 ng/mL EGF. The 3D-Assay media formulation is as follows: 500 mL DMEM/F12 (Invitrogen), containing 2% (v/v) Horse serum (Solarbio), 0.5 µg/mL, 100 ng/mL and 10 µg/mL of Hydrocortisone, Cholera toxin and Insulin, respectively (MedChemExpress), and 1% penicillin (50 U/mL)-streptomycin (50 µg/mL) (Invitrogen).

### Human breast tissue samples

Breast tissue samples were collected from the full information database of breast cancer patients in the Breast Cancer Pathology and Research Laboratory of Cancer Hospital of Tianjin Medical University (Tianjin, China), including para-cancerous tissues and breast cancer tissues diagnosed as invasive breast cancer by two senior pathologists of six patients. The research procedure of this work is in line with the ethical standards of Tianjin Medical Oncology Institute and Hospital Ethics Committee. This study was approved by the Evaluation Committee of Cancer Hospital of Tianjin Medical University (Approval No: bc2021086). And all breast cancer patients were fully informed and signed the informed consent form.

### Bioinformatics mining methods

The gene expression profiles from The Cancer Genome Atlas (TCGA) dataset and the Genotype-Tissue Expression (GTEx) project were analyzed by Gene Expression Profiling Interactive Analysis (GEPIA, http://gepia.cancer-pku.cn/index.html). The expression of PKCζ was visualized in a Boxplot [41, 42]. It is noticeable that, due to the often considerably large differences within the GTEx groups, statistically significant analysis of gene expression data may not be readily achieved for a target gene. Therefore, we carried out Gaussian normalization on the expression of genes to reduce the differences between samples within the group. After adjustment, the standard deviations within the group became smaller, yielding statistically significant *P*-values between the groups.

### Cell lines

Three stable cell lines were established from a non-tumorigenic human mammary gland epithelial cell line MCF-10 A: a HA-tagged RHBDF1-overexpressing cell line MCF-10 A/RH, an empty vector transfected control cell line MCF-10 A/GFP, and a cell line with tetracycline-inducible RHBDF1 (TetON-RH). After resistance selection, we identified the RHBDF1 expression from both mRNA (Figure S1A, C) and protein (Figure S1B, D) levels. Next, MCF-10 A/GFP and MCF-10 A/RH cell lines were cultured on reconstituted extracellular matrix proteins (Matrigel) for 4, 8, 12 days, by representative point in time of the cells formed spheroids, and we found that there was no difference in the cell masses formed by the two cell lines on the day 4, however, the difference between them was detected on the day 8 and 12 (Figure S1E). The MCF-10 A/GFP cell lines formed acinar structures on the 8th day and retained to the 12th day, evident from the presence of a central lumen revealed by confocal microscopic analysis about 90% of the spheroids surrounded by a single layer of cells (Figure S1E, F). In contrast, most of the spheroids formed by MCF-10 A/RH cells not only lacked polarized with filled lumen (75%), but also further enlarged cell masses (Figure S1E, F). These also confirmed our previous results that RHBDF1 correlates strongly with nonhollow acinar structures during morphogenesis and perturbation of epithelial apicobasal polarity [8].

### Cell invasion assay

Eight-µm pore size transwell plates were purchased from Corning (catalog #3422; State of New York, USA) covered with Matrigel (BD Biosciences). The culture medium contained 20% Horse serum in the lower chambers. The upper chamber contained established stable cells (2 × 10^4^) with serum-free media and incubated for 24 h at 37 °C. Then the cells were fixed and stained with paraformaldehyde and 1% crystal violet (Solarbio, Beijing, China), respectively for 30 min at 37 °C in darkness. The filters of the upper chambers were subjected to inverted light microscopic analyses (200X, Axiovert200M, Zeiss, Oberkochen, Germany).

### RNA isolation and qPCR

The 3D cultured acinus was dissolved by iced 2.5 mM EDTA/PBS at 4 ℃ for about 40 min, and obtained the precipitate through centrifugation. Then the total RNA was extracted by using RNeasy Plus Microkit (catalog #74043, Qiagen, Frankfurt, Germany). Reverse transcription and quantitative real-time qPCR were performed by First-strand cDNA synthesis SuperMix kit and Top Green qPCR SuperMix kit (TransGen Biotech, Beijing, China), respectively. The primer sequences were as follows: h-RHBDF1, 5’-TGCCGTGGATTGACAACTT and 5’-CACTCACAGCGGACAGGAT; h-Occludin, 5’-CATTTATGATGAGCAGCCCCC and 5’-TGCCATGGGACTGTCAACTC; h-ZO-1, 5’-CAAAAACAGCAGGCGGAGAC and 5’-AACAGGCTGAGCGGACAAAT; h-E-cadherin, 5’-CACCACGTACAAGGGTCAGG and 5’-GGGGGCTTCATTCACATCCA; h-GAPDH, 5’-GTCTCCTCTGACTTCAACAGCG and 5’-ACCACCCTGTTGCTGTAGCCAA; h-PKCζ, 5’-GGTGCGGTTGAAGAAGAATGA and 5’-TGTCGTCTGGAAGCAGGAGTG.

### Western blot analysis

Protein samples were subjected to SDS-PAGE and transferred to PVDF membranes (Millipore, Bedford, MA, USA). Primary antibodies for immunoblotting were: RHBDF1 and ZO-1 (abcam, Cambridge Science Park, UK), HA-Tag, Occludin, E-cadherin, PKCζ, p-PKCζ/λ (Thr410/403), Phospho-Tyrosine antibody, and improtin β1 (Cell Signaling Technology, CST, Boston, MA, USA), LaminB1 and GAPDH (Proteintech, Wuhan, China). And the suitable secondary antibodies were used and exposed the membranes to enhanced chemiluminescence-plus reagents (Advansta Corporation, CA, USA). Finally, they were analyzed with a Chemiluminescence imaging system (Tanon, Shanghai, China) and Gel-Pro Analyzer software V.4.0 (Media Cybernetics, MD, USA).

### Co-immunoprecipitation (co-IP)

Dissolved 3D acinus was lysed on ice by NP-40 Lysis Buffer (Beyotime, Beijing, China) containing a cocktail of phosphatase inhibitors (MedChemExpress, New Jersey, USA) and PMSF (Beyotime, Biotechnology, Haimen, China). Target proteins were isolated by using specific antibodies (PKCζ, improtin β1 or normal IgG) linked on magnetic beads (catalog #B23202, Bimake, Texas, USA), then subjected to Western blot analysis.

### Immunofluorescence staining (IF)

Established stable cells were seeded in a 15 mm glass bottom cell culture dish (NEST Biotechnology Co.LTD, Hongkong, China), then immunostained with primary antibody (PKCζ catalog #ab59364; RHBDF1 #NBP 1-59725; ZO-1 #ab216880, Abcam; HA-Tag, #2350, #3444; Occludin, #91131; E-cadherin, #14472, CST) and Alexa Fluor 488-conjugated or 568-conjugated secondary antibody (catalog #A-21202, #A10042, Invitrogen), and DAPI dye staining (Beyotime). The IF staining of mammary epithelial organoids was operated as previously described [40].

### Statistical analysis

Statistical analysis was performed by GraphPad Prism 5.0 (GraphPad Software, CA, USA). Either two-tailed Student’s t-test or one-way ANOVA was used as needed. *P-*value < 0.05 was considered statistically significant.

## Electronic supplementary material

Below is the link to the electronic supplementary material.

Supplementary Material 1

## Data Availability

The data generated during the current study are available from the corresponding author on reasonable request.
