# Supplementary figures and images for: Perturbation of mammary epithelial cell apicobasal polarity by RHBDF1-facilitated nuclear translocation of PKCζ
Source: Biol Res. 2024 Nov 24;57:90. doi: 10.1186/s40659-024-00566-2 (PMC11587606; doi:10.1186/s40659-024-00566-2)

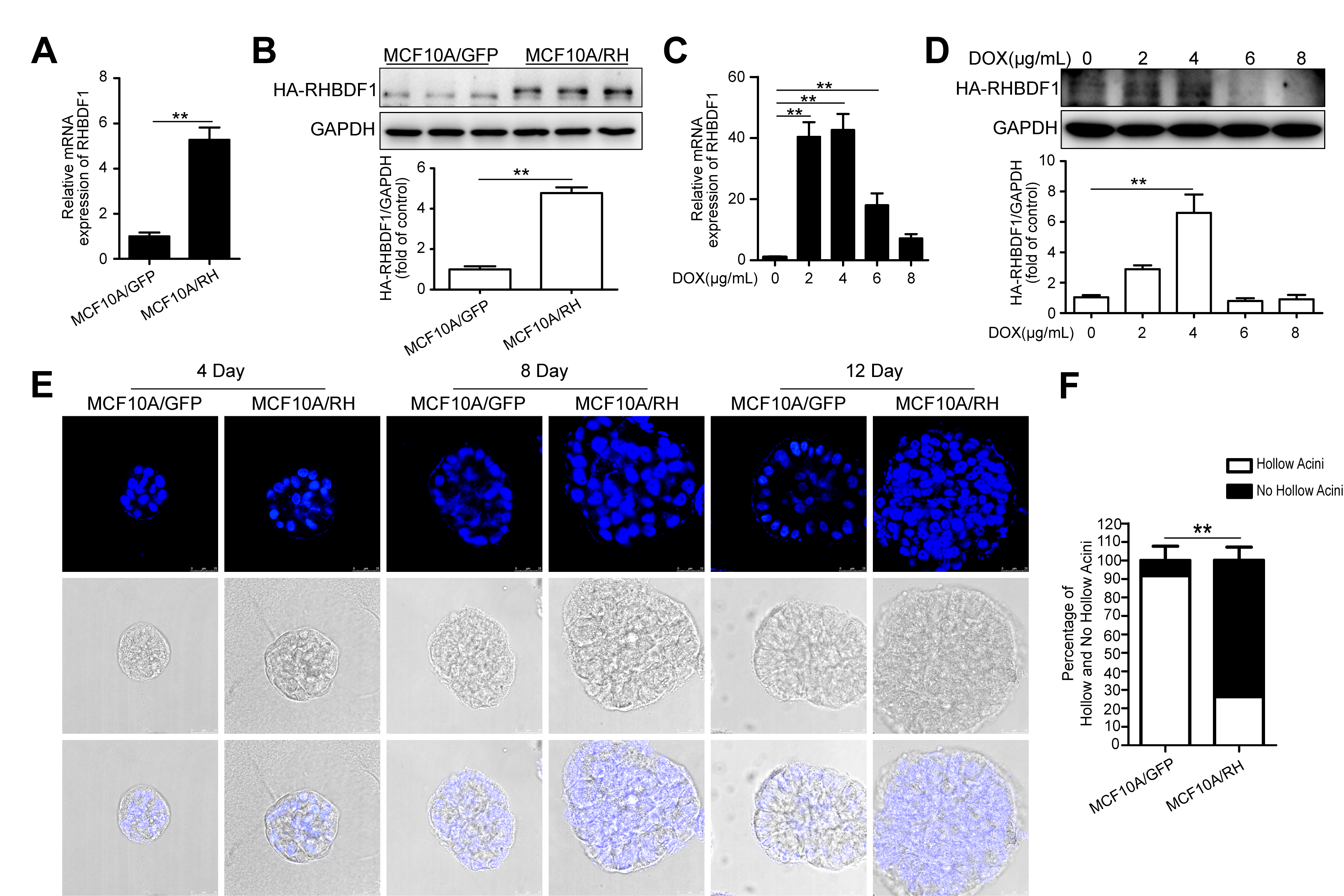

Supplement: Supplementary file 1 — Supplementary Material 1 [file 40659_2024_566_MOESM1_ESM.tif]
